# Evaluation of the feasibility of explainable computer-aided detection of cardiomegaly on chest radiographs using deep learning

**DOI:** 10.1038/s41598-021-96433-1

**Published:** 2021-08-19

**Authors:** Mu Sook Lee, Yong Soo Kim, Minki Kim, Muhammad Usman, Shi Sub Byon, Sung Hyun Kim, Byoung Il Lee, Byoung-Dai Lee

**Affiliations:** 1 Department of Radiology, Keimyung University Dongsan Hospital, 1035, Dalgubeol-daero, Sindang-dong, Daegu, 42601 Republic of Korea; 2 Division of ICT Convergence, Kyonggi University, 154-42, Gwanggyosan-ro, Yeongtong-gu, Suwon, Gyeonggi-do 16227 Republic of Korea; 3 Division of AI Computer Science and Engineering, Kyonggi University, 154-42, Gwanggyosan-ro, Yeongtong-gu, Suwon, Gyeonggi-do 16227 Republic of Korea; 4 Center for Artificial Intelligence in Medicine and Imaging, HealthHub, Co. Ltd., 623, Gangnam-daero, Seocho-gu, Seoul, 06524 Republic of Korea; 5 Human Medical Imaging and Intervention Center, 621, Gangnam-daero, Seocho-gu, Seoul, 06524 Republic of Korea

**Keywords:** Computer science, Information technology, Medical imaging

## Abstract

We examined the feasibility of explainable computer-aided detection of cardiomegaly in routine clinical practice using segmentation-based methods. Overall, 793 retrospectively acquired posterior–anterior (PA) chest X-ray images (CXRs) of 793 patients were used to train deep learning (DL) models for lung and heart segmentation. The training dataset included PA CXRs from two public datasets and in-house PA CXRs. Two fully automated segmentation-based methods using state-of-the-art DL models for lung and heart segmentation were developed. The diagnostic performance was assessed and the reliability of the automatic cardiothoracic ratio (CTR) calculation was determined using the mean absolute error and paired t-test. The effects of thoracic pathological conditions on performance were assessed using subgroup analysis. One thousand PA CXRs of 1000 patients (480 men, 520 women; mean age 63 ± 23 years) were included. The CTR values derived from the DL models and diagnostic performance exhibited excellent agreement with reference standards for the whole test dataset. Performance of segmentation-based methods differed based on thoracic conditions. When tested using CXRs with lesions obscuring heart borders, the performance was lower than that for other thoracic pathological findings. Thus, segmentation-based methods using DL could detect cardiomegaly; however, the feasibility of computer-aided detection of cardiomegaly without human intervention was limited.

## Introduction

Cardiomegaly, also known as an enlarged heart, is a symptom of cardiac insufficiency that occurs in various diseases and conditions, including high blood pressure, coronary artery disease, heart valve disease, and pulmonary hypertension. Because of its noninvasive nature, minimal radiation exposure, and economic considerations, chest X-ray imaging (CXR) is one of the most widely used medical imaging tests for early cardiomegaly detection. Recent advances in deep learning (DL) technologies and large-scale CXR database constructions have enabled significant performance improvements in computer-aided detection of cardiomegaly to a level comparable to that of radiologists^1–12^. Methods based on binary classification of the entire CXR into cardiomegaly detection via image-level label-dependent learning have dominated in the literature on DL-based automated detection of cardiomegaly. However, classification-based methods have an intrinsic limitation, as the mechanism by which DL arrives at the conclusion (i.e., the condition of anomalies in heart structure) remains obscure. Conversely, segmentation-based methods extract boundaries of the lungs and heart on CXRs to automatically calculate the cardiothoracic ratio (CTR), which is a useful index of cardiac enlargement. These approaches are more intuitive and explainable than the classification-based methods and have demonstrated promising results for limited datasets^5–12^.

The aim of this study was to determine the feasibility of explainable computer-aided detection of cardiomegaly on chest radiographs. Hence, we developed two fully automated segmentation-based methods using state-of-the-art DL models for lung and heart segmentation, and evaluated their diagnostic performance and reliability for CTR measurements using chest radiographs of normal patients and those of patients with diverse thoracic pathological conditions commonly encountered in routine clinical practice. According to the experimental results, CTRs derived from deep learning models and diagnostic performance exhibited excellent agreement with reference standards (method 1: the area under the receiver operating characteristic curve [AUC] = 0.96, *p* = 0.655; method 2: AUC = 0.95, *p* = 0.917). Performance of segmentation-based methods differed depending on the thoracic pathological conditions. When tested using chest x-ray images with lesions obscuring heart borders, the performance was lower than that for other thoracic pathological findings (method 1: AUC = 0.86, *p* = 0.003; method 2: AUC = 0.81, *p* = 0.001).

## Results

### Study participants

In total, 1000 posterior-anterior (PA) CXRs of 1,000 patients (mean age ± standard deviation [SD], 63 ± 23 years; age range 10–98 years; sex, 480 men and 520 women) were included in our study (Table 1).

**Table 1:** Data and patient characteristics.

Dataset source	Training and validation datasets for segmentation of lungs and heart			Test dataset
	JSRT dataset	Montgomery dataset	In-house dataset	In-house dataset
No. of patients	247	138	408	1,000
Mean age ± SD (years)	58 ± 14	40 ± 19	50 ± 11	63 ± 23
Age range (years)	16–89	4–89	18–95	10–98
**Sex**				
Men	119	74	184	480
Women	128	63	224	520
Unknown	–	1	–	–
Characteristics	Lung nodules (n = 154)	TB (n = 58)	PX (n = 270)	Patient with thoracic pathological findings (n = 760)
	No lung nodule (n = 93)	Normal patients (n = 80)	TB (n = 138)	Normal patients (n = 240)

*JSRT* Japanese Society of Radiological Technology, *SD* standard deviation.

### Diagnostic performance of cardiomegaly detection

**Figure 1:**
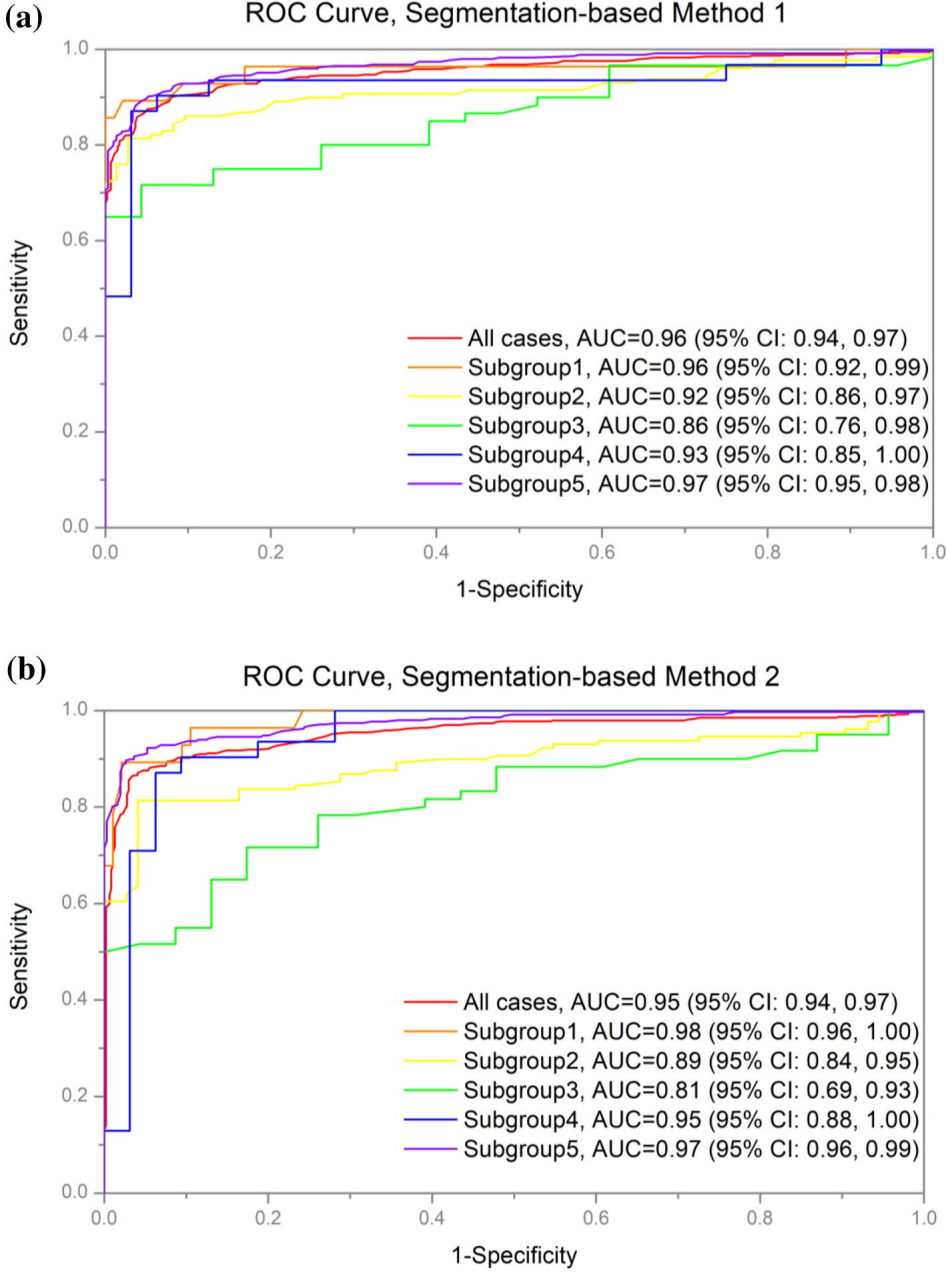
AUCs for the detection of cardiomegaly according to thoracic pathological conditions. (**a**) Segmentation-based method 1; (**b**) segmentation-based method 2. Best viewed in color. *AUC* area under the receiver operating characteristic curve.

The diagnostic performance of cardiomegaly detection is summarized in Table 2. When the entire test dataset was considered, both segmentation-based methods exhibited similar overall performance (method 1: accuracy = 91% (95% confidence interval [CI] 89, 93), sensitivity = 95% [95% CI 94, 96], specificity = 87% [95% CI 85, 89], AUC = 0.96 [95% CI 0.94, 0.97]; method 2: accuracy = 92% [95% CI 90, 94], sensitivity = 94% [95% CI 93, 95], specificity = 88% [95% CI 86, 90], AUC = 0.95 [95% CI 0.94, 0.97]). Both methods exhibited similar patterns of diagnostic performance for individual subgroups. Neither method consistently outperformed the other in all subgroups. No significant difference in performance between the methods was observed (Fig. 1). For both methods, the diagnostic performances of subgroups 2 and 3 were lower than those of other subgroups, most notably that of subgroup 3 (method 1: accuracy = 78% [95% CI 69, 87], sensitivity = 98% [95% CI 95, 100], specificity = 56% [95% CI 45, 67], AUC = 0.86 [95% CI 0.76, 0.98]; method 2: accuracy = 75% [95% CI 66, 84], sensitivity = 91% [95% CI 85, 97], specificity = 53% [95% CI 42, 64], AUC = 0.81 [95% CI 0.69, 0.93]).

**Table 2:** Diagnostic performance of detection of cardiomegaly.

Method	Category	No. of samples					Accuracy (%)	Sensitivity (%)	Specificity (%)	AUC
		TP	FP	TN	FN	n				
Segmentation-based method 1	All cases	466	66	444	24	1000	91 (89, 93)	95 (94, 96)	87 (85, 89)	0.96 (0.94, 0.97)
	Subgroup 1	25	3	91	4	123	94 (90, 98)	86 (80, 92)	97 (94, 100)	0.96 (0.92, 0.99)
	Subgroup 2	107	22	68	5	202	87 (82, 92)	96 (93, 99)	76 (70, 82)	0.92 (0.86, 0.97)
	Subgroup 3	43	17	22	1	83	78 (69, 87)	98 (95, 100)	56 (45, 67)	0.86 (0.76, 0.98)
	Subgroup 4	25	6	31	1	63	89 (81, 97)	96 (91, 100)	84 (75, 93)	0.93 (0.85, 1.00)
	Subgroup 5	314	34	288	16	652	92 (90, 94)	95 (93, 97)	89 (87, 91)	0.97 (0.95, 0.98)
Segmentation-based method 2	All cases	470	61	436	32	1000	91 (89, 93)	94 (93, 95)	88 (86, 90)	0.95 (0.94, 0.97)
	Subgroup 1	25	3	91	4	123	94 (90, 98)	86 (80, 92)	97 (94, 100)	0.98 (0.96, 1.00)
	Subgroup 2	105	24	62	11	202	83 (78, 88)	91 (87, 95)	72 (66, 78)	0.89 (0.84, 0.95)
	Subgroup 3	43	17	19	4	83	75 (66, 84)	91 (85, 97)	53 (42, 64)	0.81 (0.69, 0.93)
	Subgroup 4	28	3	29	3	63	90 (83, 97)	90 (83, 97)	91 (84, 88)	0.95 (0.88, 1.00)
	Subgroup 5	317	31	288	16	652	93 (91, 95)	95 (93, 97)	90 (88, 92)	0.97 (0.96, 0.99)

Data in parentheses indicate 95% confidence intervals.

*TP* true positive, *FN* false negative, *TN* true negative, *FN* false negative, *AUC* area under the receiver operating characteristic curve.

### Reliability of CTR calculation

We observed a high spatial overlap between the manual and automatic segmentation results for both segmentation-based methods. On validation data, the average Dice scores for the lungs and heart segmentation tasks by the segmentation-based method 1 were 0.968 and 0.955, respectively. Similarly, the average Dice scores of the segmentation-based method 2 were 0.971 and 0.957 for the lung and heart segmentation tasks, respectively.

When considering all test data, no significant differences were observed between the automatic calculation of CTRs and reference standards of both methods (method 1: *p* = 0.655; method 2: *p* = 0.917). However, individual methods exhibited different behaviors depending on thoracic pathological conditions. In method 1, the *p*-values of subgroups 1, 2, and 3 were < 0.05 (subgroup 1: *p* = 0.007; subgroup 2: *p* = 0.043; subgroup 3: *p* = 0.003). Similarly, in method 2, the *p*-values of subgroups 1 and 3 were < 0.05 (subgroup 1: *p* < *0*.001; subgroup 3: *p* = 0.002).

**Figure 2:**
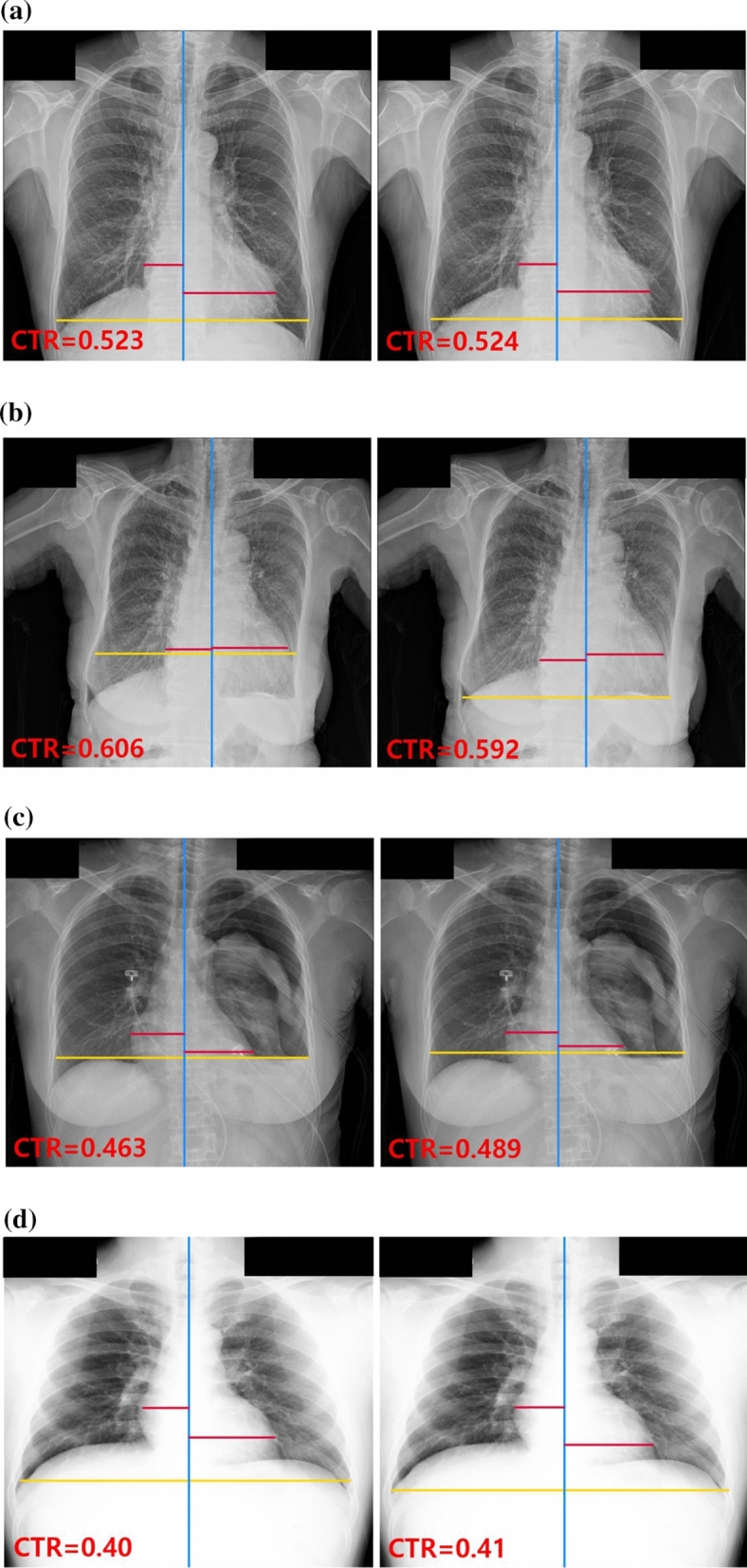
Radiographs of correctly classified examples. CTR values and three lines were generated by segmentation-based methods using deep learning models for the detection of cardiomegaly. The red lines represent the maximum transverse diameter of the left or right side of the heart, respectively; the yellow and blue lines represent the transverse thoracic diameter and the midline of the spine, respectively. For each case, the left image was generated by segmentation-based method 1, and the right image was generated by segmentation-based method 2. (**a**) PA CXR of a patient with cardiomegaly (reference standard [RS] = 0.522); (**b**) PA CXR of a patient with cardiomegaly and pleural effusion (RS = 0.611); (**c**) PA CXR of a patient with pneumothorax, pleural effusion, and left lung collapse, (RS = 0.466); (**d**) PA CXR of a patient with no finding (RS = 0.41). *CTR* cardiothoracic, *PA CXR* posterior-anterior chest X-ray.

**Figure 3:**
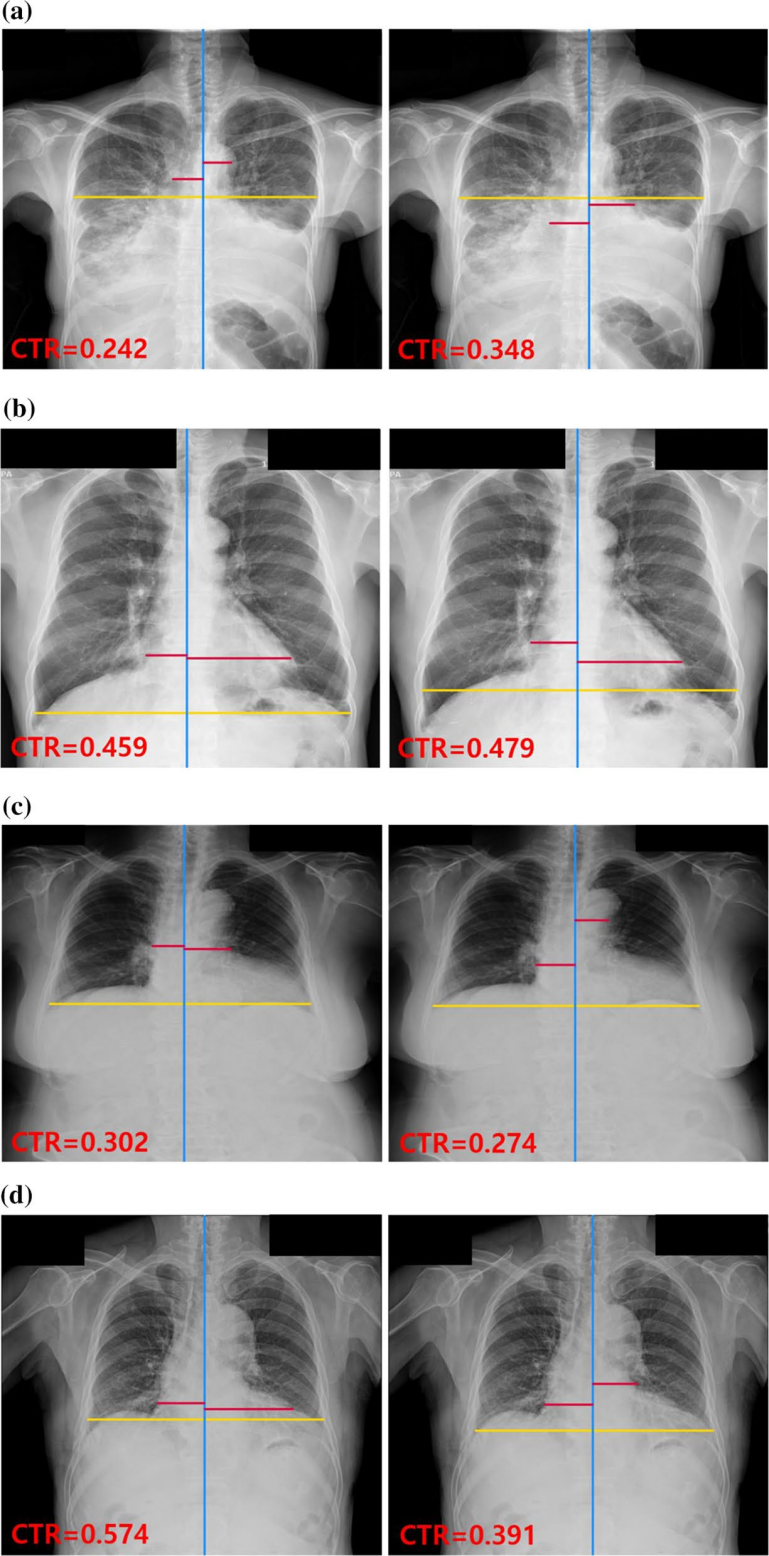
Radiographs of selected misclassified examples. CTR values and three lines were generated by segmentation-based methods using the deep learning models for the detection of cardiomegaly. The red lines represent the maximum transverse diameter of the left or right side of the heart, respectively; the yellow and blue lines represent the transverse thoracic diameter and the midline of the spine, respectively. For each case, the left and right images were generated by segmentation-based methods 1 and 2, respectively. (**a**) PA CXR of a patient with cardiomegaly, pleural effusion, consolidations in both lungs, and lesions obscuring the border of the heart (reference standard [RS] = 0.693); (**b**) PA CXR of a patient with cardiomegaly and linear atelectasis in the left lower lung (RS = 0.522); (**c**) PA CXR of a patient with cardiomegaly (RS = 0.581); (**d**) PA CXR of a patient with cardiomegaly and pleural effusion (RS = 0.639). *CTR* cardiothoracic, *PA CXR* posterior-anterior chest X-ray.

The overall mean absolute errors (MAEs) of methods 1 and 2 for the entire test dataset were 0.023 and 0.024, respectively. Similar to the diagnostic performance, the largest MAE for each method occurred in subgroup 3 (MAE ± SD, method 1: 0.058 ± 0.074; method 2: 0.059 ± 0.066). For subgroup 5, in which hard cases were excluded, the corresponding MAEs were the smallest in each method (MAE ± SD, method 1: 0.019 ± 0.024; method 2: 0.019 ± 0.023). The results are summarized in Table 3 and performance examples are presented in Figs. 2 and 3.

**Table 3:** Reliability of the cardiothoracic ratio calculation.

Method	Category	n	Mean ± SD (RS)	Mean ± SD (predicted)	95% CI for mean differences	T-value	*p*-value	MAE ± SD
Segmentation-based method 1	All cases	1000	0.506 ± 0.080	0.505 ± 0.080	(− 0.00195, 0.00310)	0.45	0.655	0.023 ± 0.033
	Subgroup 1	123	0.449 ± 0.068	0.455 ± 0.069	(− 0.01156, − 0.00190)	− 2.76	0.007	0.020 ± 0.020
	Subgroup 2	202	0.530 ± 0.083	0.522 ± 0.092	(0.00026, 0.01713)	2.03	0.043	0.035 ± 0.050
	Subgroup 3	83	0.543 ± 0.088	0.514 ± 0.100	(0.01004, 0.04909)	3.01	0.003	0.058 ± 0.074
	Subgroup 4	63	0.493 ± 0.078	0.493 ± 0.081	(− 0.00596, 0.01459)	0.84	0.404	0.025 ± 0.032
	Subgroup 5	652	0.505 ± 0.075	0.508 ± 0.075	(− 0.00456, 0.00019)	− 1.81	0.071	0.019 ± 0.024
Segmentation-based method 2	All cases	1000	0.506 ± 0.080	0.506 ± 0.078	(− 0.00263, 0.00236)	− 0.10	0.917	0.024 ± 0.032
	Subgroup 1	123	0.449 ± 0.068	0.457 ± 0.069	(− 0.01234, − 0.00346)	− 3.52	0.001	0.021 ± 0.015
	Subgroup 2	202	0.530 ± 0.083	0.524 ± 0.094	(− 0.00194, 0.01509)	1.52	0.130	0.038 ± 0.049
	Subgroup 3	83	0.543 ± 0.088	0.514 ± 0.097	(0.01113, 0.04786)	3.20	0.002	0.059 ± 0.066
	Subgroup 4	63	0.493 ± 0.078	0.496 ± 0.081	(− 0.01237, 0.00653)	− 0.62	0.539	0.025 ± 0.028
	Subgroup 5	652	0.505 ± 0.075	0.507 ± 0.072	(− 0.00443, 0.00028)	− 1.73	0.084	0.019 ± 0.023

*CI* confidence interval, *RS* reference standard, *MAE* mean absolute error, *SD* standard deviation.

## Discussion

Segmentation-based approaches to detect cardiomegaly are designed to address cardiomegaly detection tasks on chest radiographs via segmentation of the lungs and heart and subsequent calculation of the CTR. Here, we developed two fully automated segmentation-based methods using different state-of-the-art DL models for semantic segmentation (method 1: Dice = 0.968 and 0.955 for the lungs and heart segmentation tasks, respectively; method 2: Dice = 0.971 and 0.957 for the lungs and heart segmentation tasks, respectively). We observed that the CTR values derived from the DL models and diagnostic performance exhibited excellent agreement with reference standards for the entire test dataset (method 1: AUC = 0.96 [95% CI 0.94, 0.97], *p* = 0.655; method 2: AUC = 0.95 [95% CI 0.94, 0.97], *p* = 0.917). Both segmentation-based methods exhibited noticeable performance differences depending on thoracic pathological conditions. For instance, in subgroup 3, the mean difference between the automatic CTR calculations and reference standards was statistically significant at the 5% significance level in both methods (method 1: *p* = 0.003; method 2: *p* < 0.001). Consequently, the corresponding AUCs (method 1: AUC = 0.86 [95% CI 0.76, 0.98]; method 2: AUC = 0.81 [95% CI 0.69, 0.93]) were the lowest. In contrast, in subgroup 5, which did not contain any hard cases, the diagnostic performance and reliability of CTR calculations were consistent with those obtained with the entire test dataset (method 1: AUC = 0.97 [95% CI 0.95, 0.98], *p* = 0.071; method 2: AUC = 0.97 [95% CI 0.96, 0.99], *p* = 0.084). Notably, although the paired t-test results for subgroup 1 revealed significant differences between the automatic CTR calculations and reference standards for both methods, the corresponding AUCs reflected the opposite results (method 1: AUC = 0.96 [95% CI 0.92, 0.99]; method 2: AUC = 0.98 [95% CI 0.96, 1.00]). The main reason for this was that the test data in subgroup 1 were imbalanced, as only 80% of CXRs (98 out of 123) were negative. In addition, as both DL models tended to underestimate the CTRs for negative CXRs with pneumothorax (PX), the number of true negative cases increased, leading to an increase in the overall detection accuracy for subgroup 1. This also accounted for the high specificity and low sensitivity observed in subgroup 1. For subgroup 2, no significant differences were observed between the automatic CTR calculation and reference standard (*p* = 0.130), but the diagnostic performance was relatively low. This suggested that the CTRs were not underestimated nor overestimated relative to the reference standard, but the errors were large.

The overall diagnostic performance of our methods exceeded that reported by Chamveha et al.^8^ (accuracy: 68.45%, sensitivity: 75.0%, specificity: 69.5%). The AUCs when hard cases were excluded (e.g., subgroup 5, method 1: AUC = 0.97 [95% CI 0.95, 0.98]; method 2: AUC = 0.97 [95% CI 0.96, 0.99]) were comparable to those reported by Sogancioglu et al.^9^ (0.98), in which CXRs with ambiguous heart borders were excluded. The corresponding MAEs of both methods for subgroup 5 (method 1: 0.019, method 2: 0.019) were also comparable to their results (0.0135). Li et al.^6^ reported that paired differences between their DL model and reference standards for CTR measurements (*p* = 0.55) using 500 CXRs were not statistically significant, in line with our observations. They reported poor performance of their DL model for pleural effusion (PE) and fat pad of the pericardium, which belonged to subgroups 2 and 3 in our study, respectively. Gupte et al.^10^ trained the segmentation deep learning model with 1952 CXRs obtained from a public dataset released by the National Institute of Health^13^ and two private hospitals. Interestingly, they achieved a sensitivity of 96% and a specificity of 81% using the held-out test data, and a sensitivity of 87% and a specificity of 97% using the out-of-source dataset. Saiviroonporn et al.^11^ clinically evaluated a deep learning-based automatic CTR measurement on a large dataset (n = 7517). They reported that the diagnostic performance based on the automated calculation of CTRs using the DL model can provide an excellent outcome (AUC = 0.96). However, similar to our findings, they observed that it should be improved on heart diameter calculation, which is difficult to be performed because its pixel value is low, and its edges are fused with the lung borders or the thoracic spine^12^.

The methodology used in this study is distinct to segmentation-based methods used in previous work in several ways, which makes our study novel. First, segmentation of anatomical structures in chest radiographs has been extensively investigated, but only a few studies have evaluated lung boundary detection algorithms in lungs with structural deformities^14^. Thus, in an effort to process more comprehensively a wide variety of lung shapes, we manually annotated the masks of the lungs and heart of CXRs from 270 patients with PX or manifested tuberculosis (TB), which were subsequently employed as training data. Second, previous studies have only performed statistical analyses on the entire test data as a whole to evaluate the performance of their solutions. In contrast, in our work, we analyzed the overall performance of segmentation-based methods for the entire test data and their detailed behaviors depending on various thoracic pathological conditions, particularly those recognized as hard samples for automatic CTR measurements. To the best of our knowledge, this is the first study to explore the impact of individual thoracic pathologies on the effectiveness of DL-based automatic CTR measurements and application in diagnosis. Further, we harnessed state-of-the-art semantic segmentation DL models, which may be more efficient at segmenting the lungs and heart compared to the standard 2D U-Net architectures with different backbone networks used previously^6,9,11^.

This study had several limitations. First, we only considered the CXRs from patients with PX or TB to more accurately analyze abnormal lung anatomy. In general, DL models can recognize more patterns with the availability of more training data^15^. Therefore, training using a wide variety of CXRs with ambiguous lung and cardiac silhouettes because of a disease, accidents, or postsurgical alternations would enhance the generalization capability of DL models. Second, the accurate segmentation of the lungs and heart is a prerequisite for the automatic CTR calculation. Our findings were based on results obtained from two state-of-the-art DL models for segmentation. Therefore, future studies should validate whether similar results can be obtained using other state-of-the-art DL models. Third, we used training data from different institutions, but the test data were retrieved from a single institution, which may have affected the reliability of our findings.

In conclusion, segmentation-based methods using DL detected cardiomegaly with an acceptable level of performance and better interpretability. For patients with certain thoracic pathologies, such as PE or lesions obscuring the heart border, CTR calculations may be inaccurate and generate more false positive errors. Our findings suggested that the feasibility of explainable computer-aided detection of cardiomegaly without complete human intervention is limited and, thus, careful attention should be paid to patients with certain thoracic lesions.

## Methods

This study was approved by the institutional review board of the Keimyung University Dongsan Medical Center, with a waiver for written informed consent (DSMC-2021-02-021). In addition, we confirm that all methods were performed in accordance with the relevant guidelines and regulations.

### Dataset for training DL models

In total, 408 unique PA CXRs between January 2016 and December 2019 were extracted from the picture archiving and communication system repository of our hospital (mean age ± SD, 50 ± 11 years; age range 18–95 years; 184 men and 224 women). To enable processing of a variety of lung shapes, 270 PA CXRs were randomly selected from patients with PX and the remaining 138 PA CXRs were obtained from patients with TB. Digital Imaging and Communication in Medicine images were converted into lossless 24-bit gray-scale JPEG format while maintaining their original resolution and default window level settings as stored in the Digital Imaging and Communication in Medicine metadata. All the images were de-identified before analysis. Masks of the lungs and heart in CXRs were manually segmented by a board-certified radiologist (M.L.).

**Figure 4:**
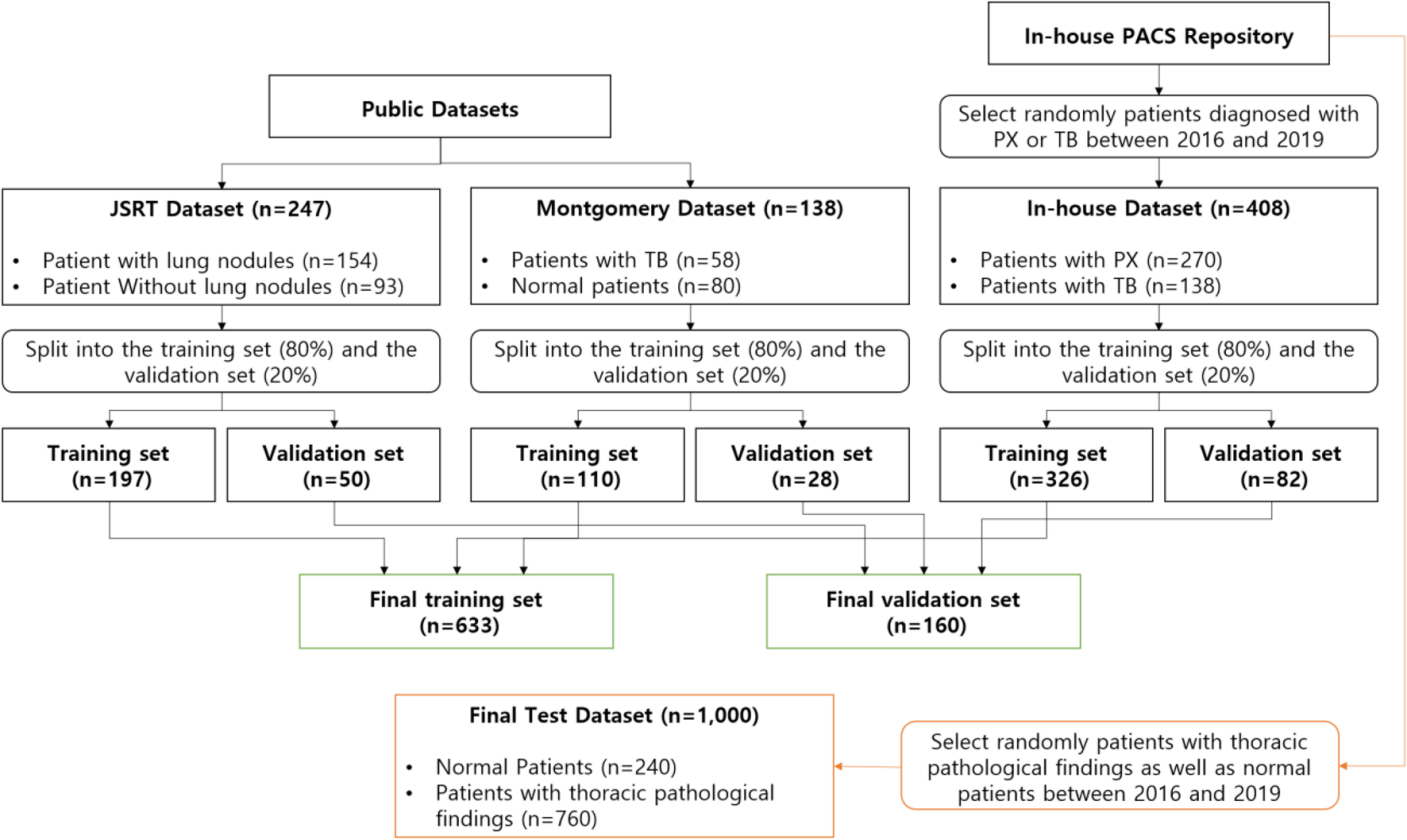
Flow chart for datasets. The final training and validation sets were used to train the DL models for lung and heart segmentation. The final test dataset was used to evaluate the segmentation-based methods using DL models. *PACS* picture archiving and communications system, *PX* pneumothorax, *JSRT* Japanese Society of Radiological Technology, *TB* tuberculosis, *DL* deep learning.

To prevent overfitting and enhance the generalization capacity of the DL models for lung and heart segmentation, CXRs obtained from two publicly available datasets were used in this study (Table 1). The Japanese Society of Radiological Technology dataset^16^ contained 247 PA CXRs, of which 154 had lung nodules (100 malignant cases, 54 benign cases) and 93 had no lung nodules. All CXRs had a resolution of 2048 × 2048 pixels and a gray-scale color depth of 12 bit. The dataset provided reference boundaries for the lungs, heart, and clavicle. The Montgomery dataset^17^ contained 138 PA CXRs, including 80 normal patients and 58 patients with TB. The resolution of CXRs was 4020 × 4892 or 4892 × 4020 pixels with a 12-bit gray-scale color depth. As the Montgomery dataset only provided pixel-wise lung mask annotations, annotations for heart masks were performed by a board-certified radiologist (M.L.). In total, 793 PA CXRs of 793 patients in different age groups were used for training DL models for lung and heart segmentation (Fig. 4).

The segmentation performance was evaluated using the Dice score that was calculated using the following formula: (2 × TP)/((TP + FP) + (TP + FN)), where TP, FP, and FN indicated the number of true positive, false positive, and false negative pixels, respectively.

### Test dataset

Additional 1000 PA CXRs were collected between January 2016 and December 2019 from our picture archiving and communication system repository to evaluate the diagnostic performance and reliability of CTR measurements of segmentation-based methods. The test dataset was carefully curated to encompass diverse cases including the CXRs with deformed lungs and/or the silhouette sign to investigate the feasibility of explainable computer-aided detection of cardiomegaly in routine clinical practice. The inclusion criteria were as follows: (1) PA CXRs and (2) no overlap of patients. Each CXR in the test dataset was reviewed and annotated by two board-certified radiologists (M.L. and S.K.). For cases of disagreement in findings or CTR measurements, consensus was achieved by discussion between the two radiologists. Consequently, the annotation information included the existence of any thoracic pathologies and the corresponding CTR value. Details of the breakdown of the test dataset are presented in Table 4.

**Table 4:** Details of the test dataset to evaluate the performance of segmentation-based methods using deep learning models for detection of cardiomegaly.

Without cardiomegaly								With cardiomegaly							
Thoracic pathological findings							Total	Thoracic pathological findings							Total
NF	CM	PX	PE	L1	L2	OT		NF	CM	PX	PE	L1	L2	OT	
**✓**							241 (24.1)		✓						291 (29.1)
		✓					44 (4.4)		✓	✓					6 (0.6)
			✓				24 (2.4)		✓		✓				53 (5.3)
				✓			4 (0.4)		✓			✓			15 (1.5)
					✓		12 (1.2)		✓				✓		11 (1.1)
						✓	64 (6.4)		✓					✓	56 (5.6)
		✓	✓				11 (1.1)		✓	✓	✓				8 (0.8)
		✓		✓			4 (0.4)		✓	✓		✓			1 (0.1)
		✓			✓		2 (0.2)		✓	✓			✓		3 (0.3)
		✓				✓	12 (1.2)		✓	✓				✓	4 (0.4)
			✓	✓			3 (0.3)		✓		✓	✓			18 (1.8)
			✓		✓		2 (0.2)		✓		✓		✓		7 (0.7)
			✓			✓	13 (1.3)		✓		✓			✓	25 (2.5)
					✓	✓	5 (0.5)		✓			✓	✓		1 (0.1)
		✓	✓	✓			2 (0.2)		✓			✓		✓	9 (0.9)
		✓	✓		✓		7 (0.7)		✓				✓	✓	3 (0.3)
		✓	✓			✓	6 (0.6)		✓	✓	✓	✓			1 (0.1)
		✓		✓		✓	5 (0.5)		✓	✓		✓		✓	1 (0.1)
		✓			✓	✓	2 (0.2)		✓	✓			✓	✓	1 (0.1)
			✓	✓		✓	4 (0.4)		✓		✓	✓	✓		1 (0.1)
			✓		✓	✓	1 (0.1)		✓		✓	✓		✓	11 (1.1)
				✓	✓	✓	1 (0.1)		✓		✓		✓	✓	2 (0.2)
									✓	✓	✓	✓	✓		1 (0.1)
									✓	✓	✓	✓		✓	1 (0.1)
									✓	✓	✓		✓	✓	1 (0.1)
Subtotal							469 (46.9)		Subtotal						531 (53.1)
Total: 1000 (100)															

Data in parentheses indicate the percentage of radiographs with respect to the total number of radiographs.

*NF* no finding, *CM* cardiomegaly, *PX* pneumothorax, *PE* pleural effusion, *L1* lesions obscuring the border of heart, *L2* lesions obscuring the border of diaphragm except PE, *OT* other common thoracic pathological findings.

In addition to the performance analysis for the whole test dataset, a subgroup analysis was performed to determine whether specific thoracic pathological conditions influenced the performance of segmentation-based methods. The test dataset was grouped into the following five overlapping subgroups: subgroups 1 (patients with PX), 2 (patients with PE), 3 (patients with lesions obscuring the heart border [e.g., right middle robe pneumonia, left lingular segment pneumonia, atelectasis, among others]), 4 (patients with lesions obscuring the diaphragm border excluding PE [e.g., right lower lobe consolidation, left lower lobe consolidation, cancer, among others]), and 5 (patients with no findings, cardiomegaly only, or other thoracic pathological findings that did not belong to the other subgroups). Interestingly, subgroups 1–4 included various hard cases, such as lungs with a deformed appearance or ambiguous cardiac silhouette.

### Deep neural network architecture for semantic segmentation

Two fully automated segmentation-based methods were developed and evaluated using different state-of-the-art DL models to minimize the derivation of biased conclusions. Method 1 used two separate XLSor^18^ models for segmenting the lungs and heart, respectively. The XLSor model consisted of three functional components: a deep convolutional neural network, a recurrent attention module, and segmentation layers. The deep convolutional neural network employed the ImageNet pre-trained ResNet-101^19^ as a backbone, and replaced the last two down-sampling layers with dilated convolution operations. The output feature map of the convolutional neural network was fed into the recurrent attention module, in which long-range contextual information was collected from all pixels to enhance pixel-wise representation capability. Finally, the segmentation layers applied multiple transpose convolution operations to the output of the attention module to generate the final segmentation masks.

The DL model of method 2 was built upon the standard U-Net^20^ architecture for multi-class semantic segmentation (e.g., lungs, heart, and background). Similar to the XLSor model, the DL model of method 2 applied self-attention modules to improve discriminative feature representation ability. The channel attention block of the attention module extracted the inter-channel relationships of the input feature map. The spatial channel block encoded the relative importance of each spatial position of the input feature map^21^. Because of the self-contained nature of the attention module, it could be located in the U-Net architecture at any point and in any number. The current implementation applied the attention modules at shallow layers of both contracting and expanding paths of the U-Net, which was determined empirically. The code for DL models of both methods is accessible in Github (https://github.com/mkmk0612/segmentation-based-cardiomegaly-detection).

### Training and validation details

Of the total data, 80% were used as training data and the remaining 20% were used as validation data for semantic segmentation. This split was conducted for three datasets, respectively, and the resulting sets were recombined for training and validation. Because of the variation in the image intensity of CXRs in the dataset, histogram equalization was applied to reduce source-dependent variance and increase the levels of contrast before feeding CXRs into the DL models for both training and testing. Furthermore, because of the variation in the image resolution of CXRs, all CXR images were rescaled into 512 × 512 pixels, which enabled retention of sufficient visual details to delineate boundaries of the lungs and heart. Since the conventional data augmentation schemes based on affine transformations (e.g., shifting, flipping, and zooming) did not improve performance in pilot trials, data augmentation was not applied. Following prediction of lung and heart masks, image post-processing (e.g., small object removal and hole filling) was performed to further improve segmentation, followed by automatic calculation of CTR.

DL models were implemented using the PyTorch framework (https://www.pytorch.org/) with a CUDA backend. The entire networks of both DL models were trained end-to-end using the stochastic gradient descent optimizer with a mini-batch size of 4. For the DL model of method 1, the initial learning rate was 0.02 and was updated using a ploy learning rate policy^18^. For the DL model of method 2, the base learning rate was set to 0.01 and subsequently decreased by a factor of 10 when the validation set accuracy stopped improving. For both DL models, the mean square error loss function was employed, and the number of iterations for training was set to 40,000 on two graphic cards (GTX Titan XP; NVIDIA, Santa Clara, CA, USA). Early stopping was applied to avoid overfitting.

### Statistical analysis

All statistical analyses were performed using Minitab software (Minitab 17.3.1; Minitab LLC, Sate College, PA, USA) and R-programming (Version 3.1.2 [2014]; R Foundation for Statistical Computing, Vienna, Austria). Diagnostic performance was evaluated in terms of accuracy, sensitivity, specificity, and AUC; the cutoff value of the CTR regarded as cardiomegaly was set to 0.5 in accordance with the regular diagnostic practice. Reliability of automatic CTR calculation was determined using MAE and the paired t-test for the test dataset. A *p*-value < 0.05 was considered to indicate a significant difference between automatic and manual calculations.

## Data Availability

The JSRT dataset used in this study is published by the Japanese Society of Radiology Technology (JSRT) and is accessible at http://db.jsrt.or.jp/eng.php, The Montgomery dataset used in this study is published by the U.S. National Institute of Health and is accessible at https://lhncbc.nlm.nih.gov/LHC-downloads/downloads.html#tuberculosis-image-data-sets.The in-house dataset that supports the findings of this study is available at HealthHub, Co. Ltd. but restrictions apply to the availability of these data, which were used under license for the current study, and thus, are not publicly available. Data are however available from the authors upon reasonable request and with permission of HealthHub, Co. Ltd.
